# Qualitative Team Formation Analysis in Football: A Case Study of the 2018 FIFA World Cup

**DOI:** 10.3389/fpsyg.2022.863216

**Published:** 2022-07-08

**Authors:** Jasper Beernaerts, Bernard De Baets, Matthieu Lenoir, Nico Van de Weghe

**Affiliations:** ^1^CartoGIS, Department of Geography, Ghent University, Ghent, Belgium; ^2^KERMIT, Department of Data Analysis and Mathematical Modelling, Ghent University, Ghent, Belgium; ^3^Department of Movement and Sports Sciences, Ghent University, Ghent, Belgium

**Keywords:** football, team formation analysis, team behavior, QTC, World Cup

## Abstract

In this paper, we explore the use of the Static Qualitative Trajectory Calculus (QTC_S_), a qualitative spatiotemporal method based on the QTC, for the analysis of team formations in football. While methods for team formation analysis in sports are predominantly quantitative in nature, QTC_S_ enables the comparison of team formations by describing the relative positions between players in a qualitative manner, which is more related to the way players position themselves on the field. QTC_S_ has the potential to allow to monitor to what extent a football team plays according to a coach’s predetermined formation. When applied to multiple matches of one team, the method can contribute to the definition of the playing style of a team. We present an experiment aimed at identifying the team formation played by Belgian national football team during the 2018 FIFA World Cup held in France.

## Introduction

In this paper, we explore the use of the Static Qualitative Trajectory Calculus (QTC_S_), a recently introduced method for describing spatial formations, for team formation analysis in football (Beernaerts et al., 2018). Team formation analysis in football might be used to increase team performance with the aim of scoring more (and conceding less) goals and therefore winning more matches.

This introductory section includes a concise overview of established methods for analyzing team formations in football. Subsequently, we briefly present the QTCs method as well as the experimental setup in section Materials and Methods. This experiment, which serves as the main part of this paper, aims at identifying the team formations played by the Belgian national football team during two matches of the 2018 FIFA World Cup. In section Results, the results of the experiment are presented. Section Discussion consists of a more thorough discussion and validation of the results, illustrating the use of the method for analyzing team performance in football.

### Team Formation Analysis in Football

This section presents an overview of the established methods used to study the spatial formation of football teams, which is referred to as “team formation analysis” in the remainder of this paper. In football, teams are reported to play in specific spatial configurations, called team formations (Kaminka et al., 2003; Kuhlmann et al., 2005). A team formation can be defined as “A specific structure defining the distribution of players based on their positions within the field of play” (Ayanegui-Santiago, 2009). While several methods for analyzing team formations have been described in various team sports (e.g., American football: Atmosukarto et al., 2013; basketball: Lucey et al., 2014; and volleyball: Jäger and Schöllhorn, 2012), this analysis can be regarded as particularly challenging in football because of the fluent and dynamic character of the game (Atmosukarto et al., 2013).

For a coach, it is important to correctly estimate the strengths and weaknesses of his or her own team. In this way, he/she can choose the most suitable team formation for a game (Carling, 2011). The selected team formation will thereby be dependent on the expected formation of the adversary team as well as the characteristics of the theoretical formation and the players that are fit to play. Aquino et al. (2019), for example, studied the effects of the applied team formation on metrics such as ball possession. Low et al. (2021) studied the impact of team formation on defensive passing behavior and collective team movements. Moreover, the manipulation of the players’ positions on the field has been described as one of the key elements of how team tactics can be applied during football matches (Rein and Memmert, 2016). The analysis of team formations in football can be performed in various ways, but often starts with a visual exploration of the data, followed by the actual detection of the formations (Stein et al., 2016, 2018). Müller-Budack et al. (2019) thereby emphasize the importance of visualizing individual situations to understand the application of team formations in football, which further illustrates the complexity of this task. Some other methods make use of various Key Performance Indicators (KPIs) to determine and analyze team formations in football (Memmert et al., 2017). These KPIs are calculated based on the positions of the players on the field. For example, Sampaio and Macãs (2012) suggested the team centroid, team entropy, a team stretch index and the surface area of the team as KPIs for team formation analysis. Going further on this, Frencken et al. (2012) added the inter-team distance, i.e., the distance between the centroids of both teams, as a key performance indicator to observe goals or shots on target during a football match.

Bialkowski et al. (2014) introduced a method for the automated detection of the type of team formation performed in a match, based on the average positions of the players on the field. They argue that, because the players are swapping positions during the game, team formation needs to be analyzed using a dynamic instead of a static ordering of players. Using data from an entire Premier League season, Lucey et al. (2013) and Bialkowski et al. (2014) found no significant difference between formations of different teams, but could detect that English Premier League teams used more offensive team formations during home games. Narizuka and Yamazaki (2019) proposed a clustering technique to compare team formations in football based on the positions of the players summarized in so-called heatmaps.

Various new methods use principles of (artificial) neural networks (McCulloch and Pitts, 1943) for sports analytics in football. Visser et al. (2001) used artificial neural networks to recognize the team formation of the opponent team by using a set of predefined reference team formations. Expanding on this, Ayanegui-Santiago (2009) suggested the inclusion of multiple relations between players for the recognition of team formations in football. For this purpose, players were divided into three groups (defenders, midfielders, and attackers) based on their role in the game and their averaged positions. Next, notions from graph theory were applied to study the team formation, thereby only including links between nodes of adjacent groups.

While most methods use quantitative metrics, Perin et al. (2013) argue that quantitative analysis alone might not be sufficient to understand the team formation of a game or an entire season. Perin et al. thereby focus on the qualitative fragmentation of a football match to obtain meaningful temporal fragments (e.g., similar defending or attacking situations) that can then be compared. Unfortunately, qualitative team formation analysis in football is at present mostly performed by human experts and is therefore rather labor-intensive (Bialkowski et al., 2014). Moreover, few studies have analyzed team formations on real size football fields, or on real football matches. Gonçalves et al. (2017); however, demonstrated that there are important differences between team tactics (and thus team formations) of matches played on a football field with a reduced size and games played on a full field, suggesting that research should be conducted on official football matches. As in other sports disciplines, mixed methods—which combine both quantitative and qualitative principles—have been gaining importance in sports analytics for football in recent years (Anguera et al., 2017). There are, however, only a limited number of mixed methods that are specifically aimed at analyzing and defining team formation in football (Frencken et al., 2012; Sampaio and Macãs, 2012; Aquino et al., 2019). These methods primarily combine quantitative performance metrics (e.g., ball possession, running speeds or number of shots, and goals) with qualitative observations of the performed team formations in order to evaluate the effectiveness of the played team formation(s). In the remainder of the paper, however, we propose a method that primarily aims at detecting the performed team formation rather than evaluating its effectiveness or impact on quantitative performance metrics.

## Materials and Methods

In this section, we briefly discuss the proposed methodology for the analysis of sports team formation in football. The theoretical basis was previously introduced by Beernaerts et al. (2018), but was never applied on real football data with the aim of obtaining knowledge on team formation behavior, necessitating a validation of the results with findings of football experts. More importantly, this section therefore focuses on the experimental setup.

### Static Qualitative Trajectory Calculus

**Figure 1:**
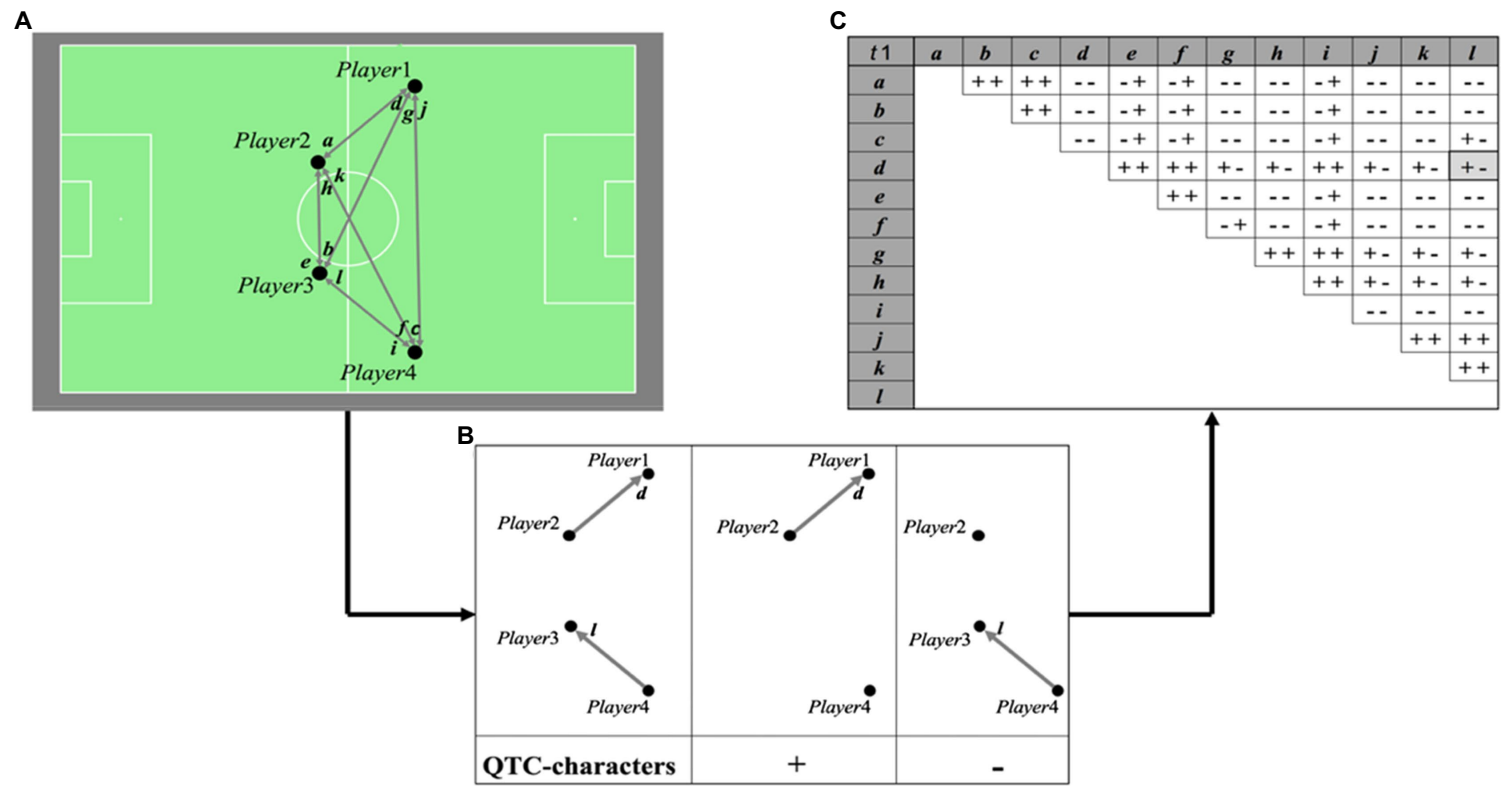
A formation of four players (POs) on a football field at t1 and the vectors between them **(A)**. The construction of the Qualitative Trajectory Calculus (QTCs)-relations between two vectors *d* and *l*, consisting of the QTC_S_-relation of vector *d* with respect to the starting point of vector *l* and of the QTC_S_-relation of vector *l* with respect to the starting point of vector *d*. If the vector moves away from the starting point of the other vector, the QTC_S_-relation is denoted by “+,” if the movement is toward the marker, the QTC_S_-relation is denoted by “−.” If the movement is neither away nor toward the marker (thus perpendicular to the connecting line between the two starting points), the QTC_S_-relation is denoted by “0” **(B)**. The QTC_S_-matrix describing the full formation of the four players, including all relations between all the vectors (**C**; Reproduced from Beernaerts et al., 2018, with permission from icSPORTS).

QTC is a qualitative calculus for describing spatiotemporal relations between two or more Moving Point Objects (MPOs; Van de Weghe and De Maeyer, 2005). While QTC typically describes movement between multiple objects, it was extended to a new variant named the QTC_S_ by Beernaerts et al. (2018). While multiple variants of the dynamic QTC exist, the QTC_S_ method presented in this paper makes use of the principles of the most basic dynamic variant QTC_B_ (Van de Weghe and De Maeyer, 2005). QTC_S_ describes the static formation of Point Objects (POs) in a qualitative manner and can be used to describe the spatial formation of players in football, which is shown for a basic example in Figure 1. Its use for analyzing team formations of (parts of) football teams was demonstrated by Beernaerts et al. (2018) by means of some basic examples.

By constructing a QTC_S_-matrix for each team formation of interest (i.e., timestamp), QTC_S_ can be used to describe the team formation at different moments during a match, by calculating the distance between the QTC_S_-representation at each moment and the QTC_S_-representation of a series of reference team formations, thereby assigning the reference team formation with the lowest distance (the highest similarity) as a label for the team formation of that respective timestamp. The distance between two QTC_S_-matrices can be calculated by summing up the pairwise distances between the corresponding elements (QTC_S_-relations), thereby using the conceptual distance between QTC-relations (Van de Weghe and De Maeyer, 2005). By dividing the total distance by the maximal possible distance (depending on the matrix dimensions), the relative distance is calculated. For ease of understanding, the relative distance is recalculated to a similarity value between 0 and 1. One could argue that QTC_S_, a method that originated from the geographical research domain, can therefore be considered a mixed method for analyzing team formations in football, as it combines qualitative spatial reasoning with a quantitative distance calculation.

**Figure 2:**
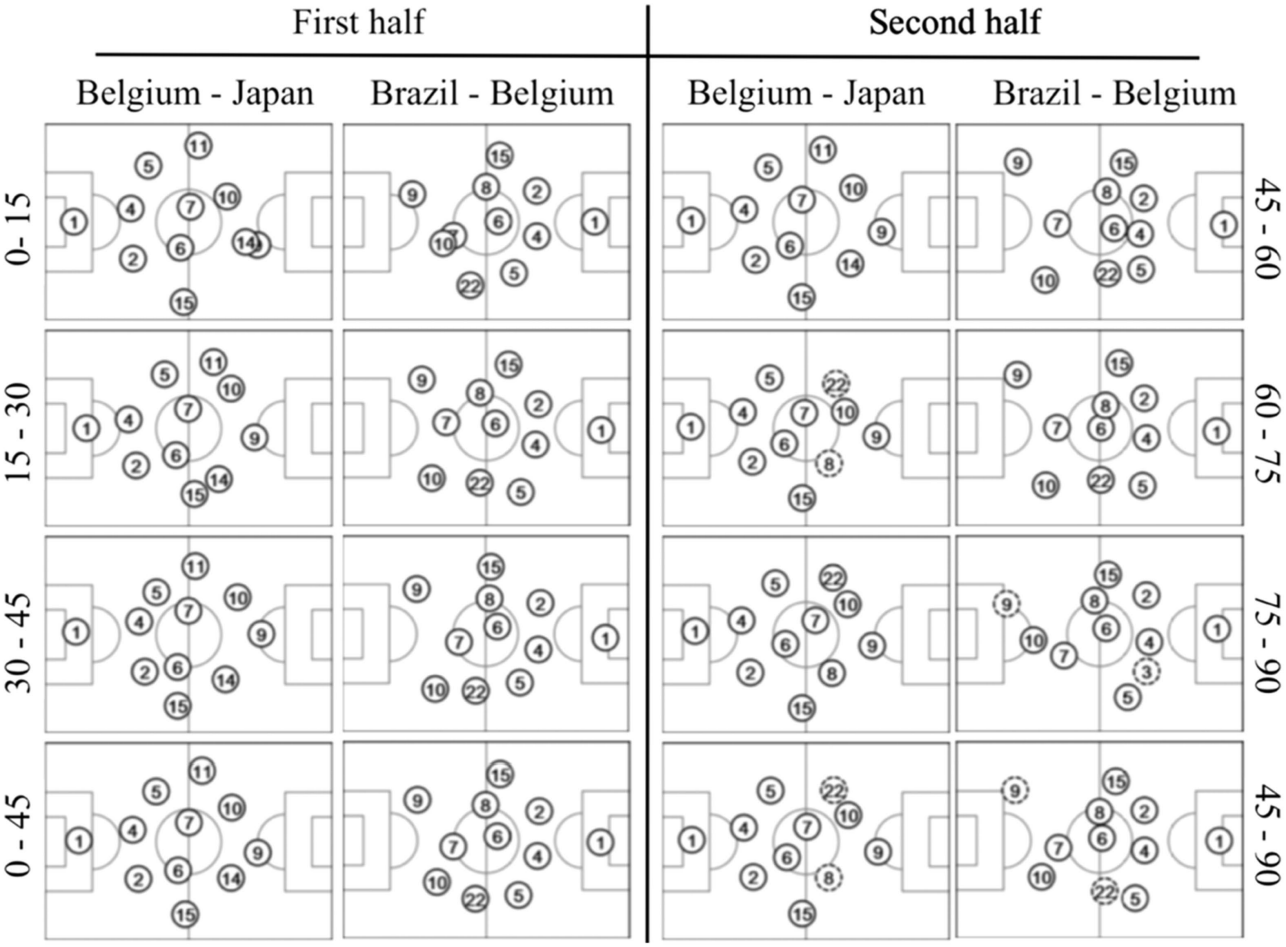
Actual performed team formations of the Belgian national football team.

**Figure 3:**
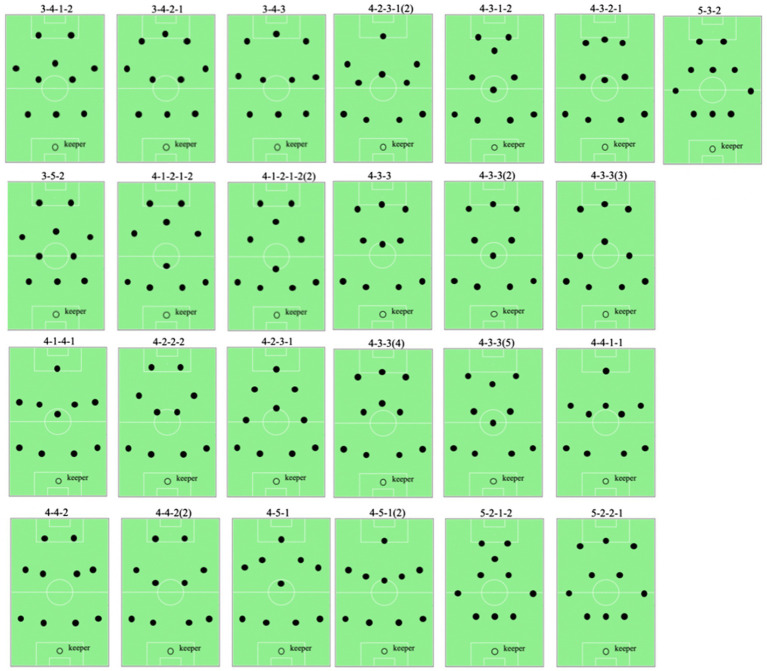
The 25 reference team formations used for the experiment. The data used in this figure were taken from FifaUTeam (2016).

The experiment aims at defining the team formation of the Belgian national football team during two football matches (Belgium—Japan and Brazil—Belgium). Most studies are limited to the analysis of the impact of different team formations (labeled manually by football experts) on different football metrics. In this experiment, however, we aim at investigating the use of QTC_S_ as a method for the (automated) detection (thus labeling) of team formations. The data was extracted from the official FIFA match reports (FIFA, 2018), which are publicly available and consist of a theoretical team formation (filed by the coach before the start of the game) and a series of actual positions of the players averaged over every 15, 45, and 90 min of both matches, visualized in Figure 2. Unfortunately, no generally accepted dataset of reference football team formations has been reported in literature (Football Bible, 2014; Wilson, 2014). Such a dataset is required for labeling the theoretical and actual performed team formations. Moreover, every coach or football analyst has his/her own interpretation of each team formation, based on personal experiences or expectations of specific player roles for that formation (Wilson, 2014). For these reasons, the reference team formation dataset was derived from the popular football simulation game FIFA 2016, as it includes a top-down view of 25 standard team formations (Figure 3), which are well-known by millions of people and are documented rigorously (FifaUTeam, 2016).

All 11 players are included in the analysis, where the keeper can be seen as a static point, fixating the rotation of the team formation (Beernaerts et al., 2018). Indeed, the goalkeeper will always be positioned in front of his own goal, thus with an almost identical orientation with respect to the rest of the players of his team. As players tend to switch roles and positions during the game (Bialkowski et al., 2014), it is necessary to include permutations of the QTC_S_-matrices when calculating the distance between the team formation at a certain moment and the different reference team formations. Calculating all permutations, however, requires 39.916.800 iterations of the distance calculation. To reduce the complexity of the experiment, four players were chosen as “fixed” based upon common sense, thereby reducing the number of players to permute to 7 and the number of permutations to 5,040. Indeed, assuming that a football team always plays with one goalkeeper and at least three defenders, these players can be omitted. This corresponds with the reference team formations dataset, which does not contain a team formation without goalkeeper or with less than 3 defenders. Furthermore, defensive players are characterized by a high level of positional rigidity (Gonçalves et al., 2014), meaning that position switches are not likely to occur.

## Results

**Figure 4:**
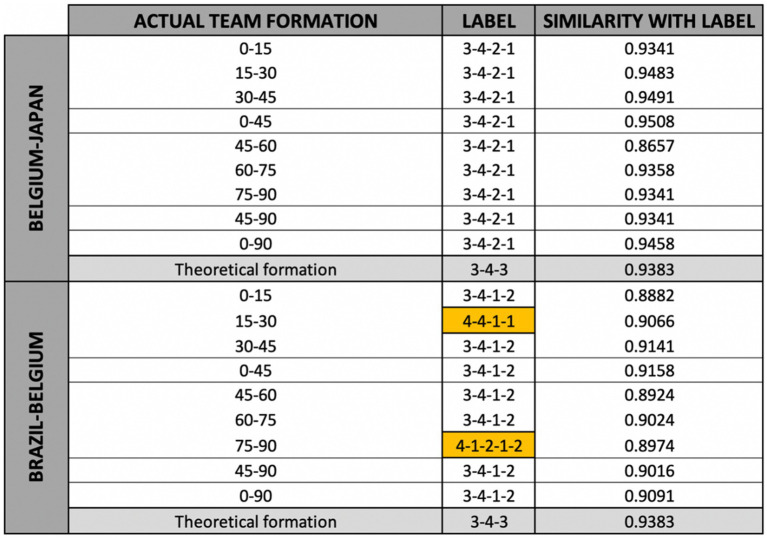
Results of the experiment showing the reference team formation with the highest similarity for each of the actual performed team formations of the Belgian national football team during two matches of the 2018 FIFA World Cup.

Figure 4 displays the results of the experiment for the matches Belgium-Japan and Brazil-Belgium. For each of the actual performed team formations included in the official FIFA match reports (nine per match), the most similar reference team formation is shown (column “label”) along with the similarity (1—distance) with that reference team formation (column “similarity with label”). While the theoretical team formation is a 3-4-3 formation for both matches, this formation is never detected as an actual performed team formation. Instead, small variants of this team formation are reported to have occurred. In the match against Japan, for example, the highly similar 3-4-2-1 formation was played by the team for all the considered time intervals. In fact, the 3-4-2-1 formation can be seen as a variant of the more general 4-3-3 formation, the only difference being that the two flank midfielders play a bit higher (i.e., more toward the opponent’s goal) and the two flank attackers play a bit closer to the middle attacker (see Figure 3). In the match against Brazil, the team most commonly played according to a 3-4-1-2 formation, another variant of the 4-3-3 formation. In this formation, the flank midfielders play a bit higher and the flank attackers play a bit closer to the center of the field, while the middle attacker plays significantly lower. In fact, the latter is the only important difference between the 3-4-1-2 and the 3-4-2-1 formations, which are both similar to the 3-4-3 formation. More remarkably, there are two time intervals (minutes 15–30 and 75–90) during the match against Brazil for which reference team formations with four defenders are detected as most similar (shown in yellow in Figure 4).

## Discussion

**Figure 5:**
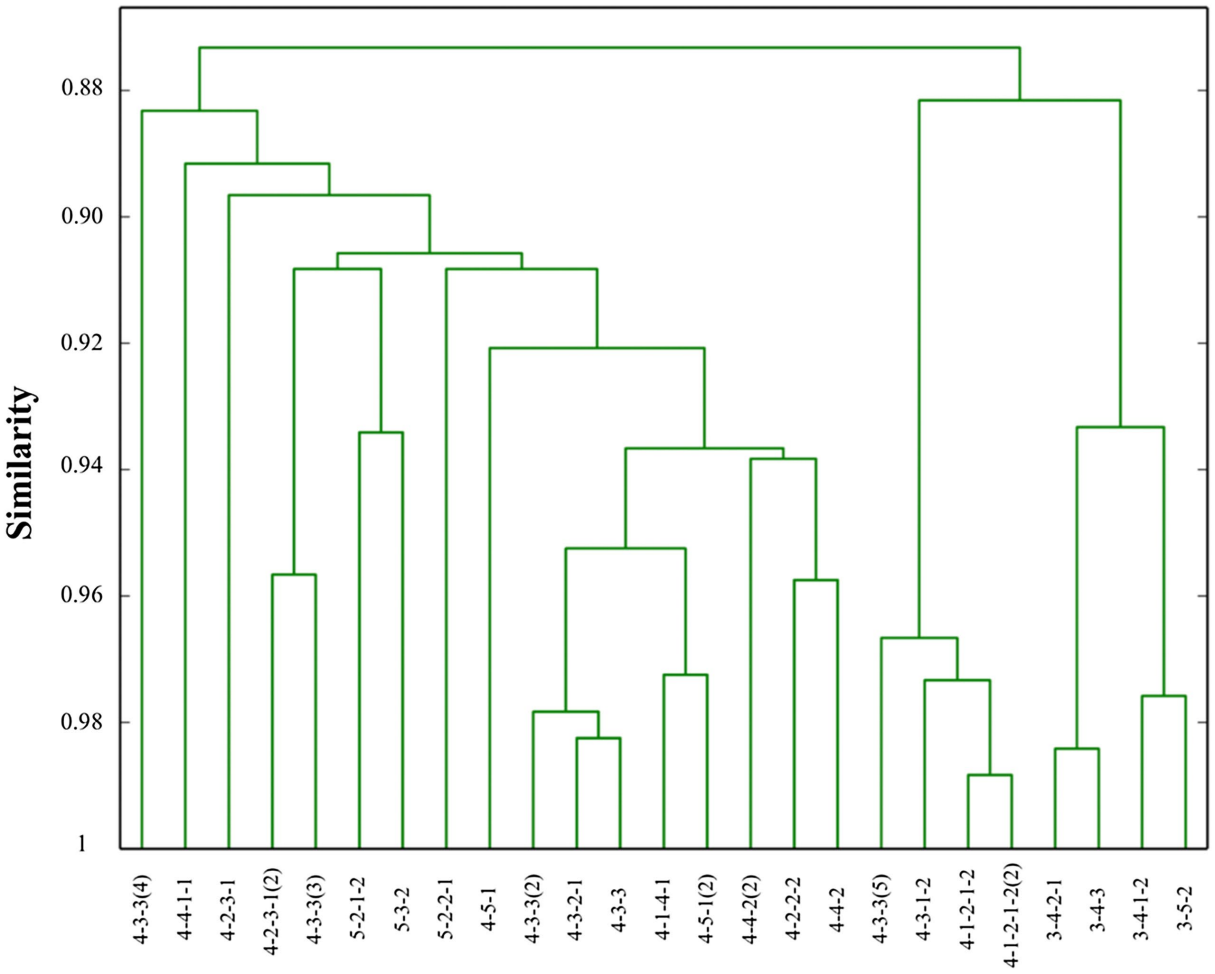
Hierarchical clustering (UPGMA) of the 25 reference team formations.

Although the use of QTC_S_ for the analysis of team formations in football has been reported (Beernaerts et al., 2018), it is important to validate the results of this specific experiment. This section therefore focuses on the validation of the experimental results, as a more detailed methodological discussion of QTC_S_ with respect to other established methods for team formation analysis can be found in Beernaerts et al. (2018). To get a good understanding of the different reference team formations, they are compared using QTC_S_. For this purpose, the distances between the QTC_S_ representations of each pair of the 25 reference team formations are calculated and stored in a distance matrix, using the method presented in section Static Qualitative Trajectory Calculus. The resulting distance matrix is used as input for the hierarchical clustering (UPGMA; Sokal and Michener, 1958) presented in Figure 5.

As Feuerhake (2016) argues, the validation of team formation analysis in football is a challenging task, mainly because of the lack of a good ground truth. As a first step of validation, it can be noted that the coach filed a 3-4-3 formation for both matches, which is very similar to the 3–4-3 variants that were detected for the whole match against Japan (3-4-2-1) and for the most part of the match against Brazil (3-4-1-2). Subsequently, match reports published by (objective) football experts in popular media can be used for further validation. In general, these reports mention that the Belgian national team played according to a 3-4-3 formation during the 2018 FIFA World Cup:

“*Belgium, during qualifying for this tournament and at the World Cup itself, have played almost exclusively in an adventurous 3-4-3 formation that packs the pitch with attackers and seeks to dominate the ball*.”—Hailsey (2018)

“*And unlike the great, gritty 1980s’ Devils, this vintage plays gorgeous football…because Martinez has fielded all his creators, in a 3-4-3 formation.*”—Financial Times (Kuper, 2018)

For the match against Japan, several popular sports media reported a 3-4-2-1 formation of the Belgian national football team (ESPN, 2018; Transfermarkt., 2018), similar to the results of the experiment (see Figure 4). Other media, however, reported that the Belgian national football team played according to the more general 3-4-3 formation during the match against Japan:

“*Belgium started in a 3-4-3 and many senior players returned to the starting line-up from their game against England. Japan lined up in a 4-2-3-1.*”—Business Standard (2018)

However, this match report includes a visual overview of what they refer to as the 3-4-3 formation (Business Standard, 2018). From this visual overview, it can be seen that this team formation much more resembles the 3-4-2-1 reference team formation than the 3-4-3 reference team formation that is used in this use case (see Figure 3). Indeed, from the visual overview it can be seen that the flank midfielders play higher and the flank attackers closer to each other than in the 3-4-3 reference team formation. For this reason, it can be assumed that in their tactical analysis, the analysts do not distinguish between the different variants of the 3-4-3 formation shown in Figure 3. Their report can therefore be seen as a positive validation of the results shown in Figure 4, especially considering the large number of reference team formations.

For the match against Brazil, various popular sports media reported that the Belgian national football team alternated between a defense with three defenders (in ball possession) and four defenders (when defending), in correspondence with the results of the experiment. Indeed, during the match against Brazil the team formation of the Belgian national football team differed quite a lot from the team formations of their previous matches:

“*On one hand, Belgium coach Roberto Martinez stunned Brazil by using an entirely unexpected system, deploying key players in new roles and shifting smoothly between a three-man defence and a back four.*”—Independent Newspaper (Cox, 2018)

“*Head coach Roberto Martinez brought Marouane Fellaini and Nacer Chadli into Belgium’s starting line-up, with Dries Mertens and Yannick Carrasco dropping to the bench. They continued with their defensive back three when in possession, but often converted to a back four when defending.*”—The Coaches Voice (2018)

Other reports go beyond describing the changing number of defenders and describe the adjustments made to the team formation, thus including all the players:

“*As can be seen above, this structure occurred when De Bruyne dropped deep to create a 4-3-1-2 deep block in order to fashion a 4v3 against Neymar, Coutinho, and Marcelo.*”—Outside of The Boot (Arvind, 2018)

Unfortunately, the 4-3-1-2 formation mentioned by Arvind (2018) is not included in the results shown in Figure 4. The 4-1-2-1-2 formation which is detected as label for the time interval between the 75th and the 90th minute, however, is very similar to the reported 4-3-1-2 formation (see Figure 5). Looking at the time interval between the 15th and the 30th minute, the 4-4-1-1 formation label cannot be explained by using the hierarchical clustering tree. Indeed, the 4-4-1-1 formation seems to have rather large distances with the expected formations (i.e., the 4-3-1-2 formation). Remarkably, the similarity with the label is rather low for this time interval (15th—30th minute) when compared to the other labels of this match and especially with the labels of the match against Japan. This means that the team did not play strictly according to one of the reference team formations. The importance of the label for this time interval should therefore not be overestimated. The strict definition of the time interval can be one of the reasons for this, as during a rather long period of 15 min a team can play according to multiple actual performed team formations that better match with one of the reference team formations. During these longer time intervals, however, the players’ coordinates are averaged, meaning that these labels with higher similarities might not be detected.

This experiment illustrates the use of QTC_S_ for team formation analysis in football. One of the main benefits of this qualitative method is the fact that it maps the topology of the team formation. This allows to better deal with scale differences or small changes (or errors) in the positional data of the players, as discussed in Beernaerts et al. (2018).

Whereas the experiment presented in this paper illustrates the use of QTC_S_ for team formation analysis in football by including an in-depth evaluation of its result for two football matches, the inclusion of more matches is required to use QTC_S_ as a method for systematic observation in football (Anguera et al., 2019). This would eventually make it possible to define the playing style of a football team based on QTC_S_. Besides the application of the method to a larger number of football games, future work might also include, among others, the assessment of real-time applications of the QTC_S_ method in football. A first example of this could be the evaluation of the performed team formation(s) during the first half of the match. This might allow coaches to give feedback to the players during the halftime break, in an attempt to optimize team performance during the second half of the match. Indeed, as the calculations for the experiment of this paper were performed on a dual-core (2.1 Ghz) laptop with 4 Gigabyte of RAM memory and with a run-time of less than an hour, computers or servers with higher computational power could allow for such applications in the future. Additional measures, such as the calculation and the storage of QTC_S_ representations of the reference team formations prior to the match, could further reduce complexity and speed up calculations.

## Conclusion

The experiment described in this paper demonstrates the capabilities of the QTC_S_ for analyzing team formations in real football. More specifically, the experiment was aimed at defining the team formations played by the Belgian national football team during two matches at the 2018 FIFA World Cup. From match reports published in popular media, it is apparent that QTC_S_ can be used for the (automated) detection of the team formation adopted during different phases of the game.

## Data Availability Statement

The raw data supporting the conclusions of this article will be made available by the authors, without undue reservation.

## Author Contributions

All authors listed have made a substantial, direct, and intellectual contribution to the work and approved it for publication.

## Funding

This research was funded by the Research Foundation Flanders, grant number 11ZN118N.

## Conflict of Interest

The authors declare that the research was conducted in the absence of any commercial or financial relationships that could be construed as a potential conflict of interest.

## Publisher’s Note

All claims expressed in this article are solely those of the authors and do not necessarily represent those of their affiliated organizations, or those of the publisher, the editors and the reviewers. Any product that may be evaluated in this article, or claim that may be made by its manufacturer, is not guaranteed or endorsed by the publisher.
